# Vimentin contributes to epithelial-mesenchymal transition cancer cell mechanics by mediating cytoskeletal organization and focal adhesion maturation

**DOI:** 10.18632/oncotarget.3862

**Published:** 2015-04-18

**Authors:** Ching-Yi Liu, Hsi-Hui Lin, Ming-Jer Tang, Yang-Kao Wang

**Affiliations:** ^1^ Institute of Basic Medical Sciences, National Cheng Kung University, Tainan, Taiwan; ^2^ Department of Physiology, National Cheng Kung University, Tainan, Taiwan; ^3^ Department of Cell Biology and Anatomy, National Cheng Kung University, Tainan, Taiwan; ^4^ Center of Neurotrauma and Neuroregeneration, Taipei Medical University, Taipei, Taiwan

**Keywords:** vimentin, epithelial-mesenchymal transition (EMT), microtubule, focal adhesions, cell stiffness

## Abstract

Modulations of cytoskeletal organization and focal adhesion turnover correlate to tumorigenesis and epithelial-mesenchymal transition (EMT), the latter process accompanied by the loss of epithelial markers and the gain of mesenchymal markers (e.g., vimentin). Clinical microarray results demonstrated that increased levels of vimentin mRNA after chemotherapy correlated to a poor prognosis of breast cancer patients. We hypothesized that vimentin mediated the reorganization of cytoskeletons to maintain the mechanical integrity in EMT cancer cells. By using knockdown strategy, the results showed reduced cell proliferation, impaired wound healing, loss of directional migration, and increased large membrane extension in MDA-MB 231 cells. Vimentin depletion also induced reorganization of cytoskeletons and reduced focal adhesions, which resulted in impaired mechanical strength because of reduced cell stiffness and contractile force. In addition, overexpressing vimentin in MCF7 cells increased cell stiffness, elevated cell motility and directional migration, reoriented microtubule polarity, and increased EMT phenotypes due to the increased β1-integrin and the loss of junction protein E-cadherin. The EMT-related transcription factor slug was also mediated by vimentin. The current study demonstrated that vimentin serves as a regulator to maintain intracellular mechanical homeostasis by mediating cytoskeleton architecture and the balance of cell force generation in EMT cancer cells.

## INTRODUCTION

Breast cancer is a common type of cancer in females. Its prognosis and gene profiles are divided into four stages with various molecular marker expressions: the normal-like phenotype (a marker expression similar to normal breast tissue); the luminal phenotype (estrogen receptor (ER)-positive, and epithelium markers with cytokeratin CK8/18 expression) ER-negative tumors (with ErbB2 oncogene overexpression); and basal-like phenotype (epidermal growth factor receptor and basal cytokeratin CK5/6 and CK17 expression) [1, 2]. In the basal-like stage, cancer cells increase the level of cancer malignancy by using a reversible epithelial-mesenchymal transition (EMT) process [3], which can also be found in tissue repair, as well as in tissue fibrosis and cancer progression [4-6]. In addition, EMT allows a polarized epithelial cell to undergo multiple biochemical changes that enable it to assume a mesenchymal cell phenotype. Compared with normal epithelial cells, EMT cells display an enhanced migratory capacity, invasiveness, resistance to apoptosis, and an increased production of extracellular matrix (ECM) components [4, 5]. Although secondary epithelial cells can transform into cancer cells, they lose their polarity and detach from the basement membrane. These cells are expressed at the invasive fronts of primary tumors, and they can further change the composition of ECM components to promote cancer cell metastasis through circulation and form secondary tumor nodules on remote organs through the reversed mesenchymal-epithelial transition process [4]. However, EMT is not a mechanism that forms fibroblast-like cells; it is a process that results in cancer cell migration, invasion, and metastasis [4, 7]. Various biomarkers have been screened to demonstrate the EMT process, including the loss of E-cadherin, ZO-1, cytokeratin, as well as the upregulation of matrix metalloproteinase, fibronectin, α-smooth muscle actin, vimentin, snail, and slug [7].

Vimentin is a 57 KD, type III intermediate filament that is found in the mesenchymal cells of various types of tissue during their developmental stages, and that maintains cell and tissue integrity [8]. Intermediate filaments share common structures: the central rod domain, head domain at N-terminal, and the tail domain at C-terminal [9]. Switching expression types of intermediate filaments is associated with malignancy. For example, cytokeratin is downregulated and mesenchymal marker vimentin replaces cytokeratin in malignant breast cancer cells [10, 11]. Vimentin is highly expressed in high-grade ductal breast carcinoma or in tumors with low ER levels, but not in types of lobular carcinoma [10-12]. By using a treatment of Withaferin-A (WFA) (i.e., a bioactive compound with anticancer properties), cancer malignancy is substantially reduced in vimentin-expressing cancer cells. The expression of vimentin leads cancer cells more sensitive to WFA treatment, which induces cell apoptosis [13]. Vimentin is associated with cancer invasion and poor prognosis in numerous types of cancers, including breast cancer, prostate cancer, melanoma, and lung cancer, and serves as a potential target for cancer therapy [14, 15]. Though vimentin expression is found in later cancer stages and correlates to malignancy, the role of vimentin in the regulation of cancer malignancy is unclear.

During transformation, focal adhesion stability and cytoskeleton distribution are impaired. Changes in cytoskeleton organization and focal adhesion turnover are correlated to the changes in cancer cell motility and invasiveness during tumorigenesis [16, 17]. As a key regulator of focal adhesion and mechanotransduction, it has been shown that β1-integrin plays a critical role in cancer progression [18, 19]. After β1-integrin binds with its specific ECM, it is activated, clustered, and further recruits focal adhesion proteins as focal adhesion kinase (FAK) and paxillin to form naïve adhesions. After the initial adhesion, additional focal adhesion proteins, such as talin and vinculin, are recruited to form stable mature focal adhesions and to connect to actin cytoskeleton bundles. This focal adhesion maturation enhances the strength between focal adhesions and actin cytoskeletons, further generates cell contractile force, and maintains cellular mechanical strength, cell tension, and cell stiffness [16, 20, 21]. In addition to actin binding to focal adhesions, vimentin also connects to focal adhesions through filamin A [22]; which is a scaffold protein that tethers membrane receptors, such as integrin and actin cytoskeleton, and stabilizes three-dimensional actin web structures [23]. Previous *in vitro* studies have demonstrated that the knockdown of vimentin impairs cell attachment, migration, and invasion in breast and colon cancer cell lines [24]. The functions of vimentin contribute to the construction of cytoskeleton architecture within cells by interacting with microfilaments and microtubules, generating cellular mechanical strength. The *in vitro* studies that used fibroblasts have demonstrated that depletion or disruption of vimentin reduces cell stiffness [25]. By overexpressing oncogenes SV 40T and c-Myc, vimentin is reorganized, increases its fiber width, and elevates cell stiffness [26]. Unlike other types of cytoskeletons that directly contribute to cell contraction, extension, and mechanical strength, vimentin can sustain large amounts of deformation and stress and maintain cell integrity [27].

During the progression of cancer, affected tissue were demonstrated to be more rigid than normal tissue, both in clinical detection of cancer patients and in *in vivo* studies [28, 29]. Vimentin was found to be sensitive to various levels of substratum stiffness, responding through the biphasic changes of the soluble and insoluble fraction ratio in hMSC, HUVEC, and NIH 3T3 cells [30]. The loss of vimentin in mouse embryonic fibroblast cells decreased their cell stiffness homeostasis, particularly when MEFs were seeded on soft substrates [31]. Therefore, we investigated the role of vimentin during EMT-related cancer progression. To clarify how vimentin contributed to EMT-related tumorigenesis and its role in cytoskeleton coordinated mechanotransduction, we performed different stages of breast cancer cells to evaluate EMT-induced tumorigenesis and mechanotransduction. Through the application of small interfere (si) and small hairpin (sh)-RNA in MDA-MB 231 cells, we were able to knock down vimentin and investigated its functional role in cell mechanics and cancer progression. In addition, overexpression of vimentin in vimentin-negative MCF7 cells demonstrated the role of vimentin in cancer progression. In particular, this study demonstrated that vimentin plays a crucial role in maintaining cytoskeleton architecture and cellular mechanical strength, as well as mediates the organization of microtubule polarity and induces cancer cell malignancy.

## RESULTS

### Vimentin expression contributes to breast cancer development

**Figure 1:**
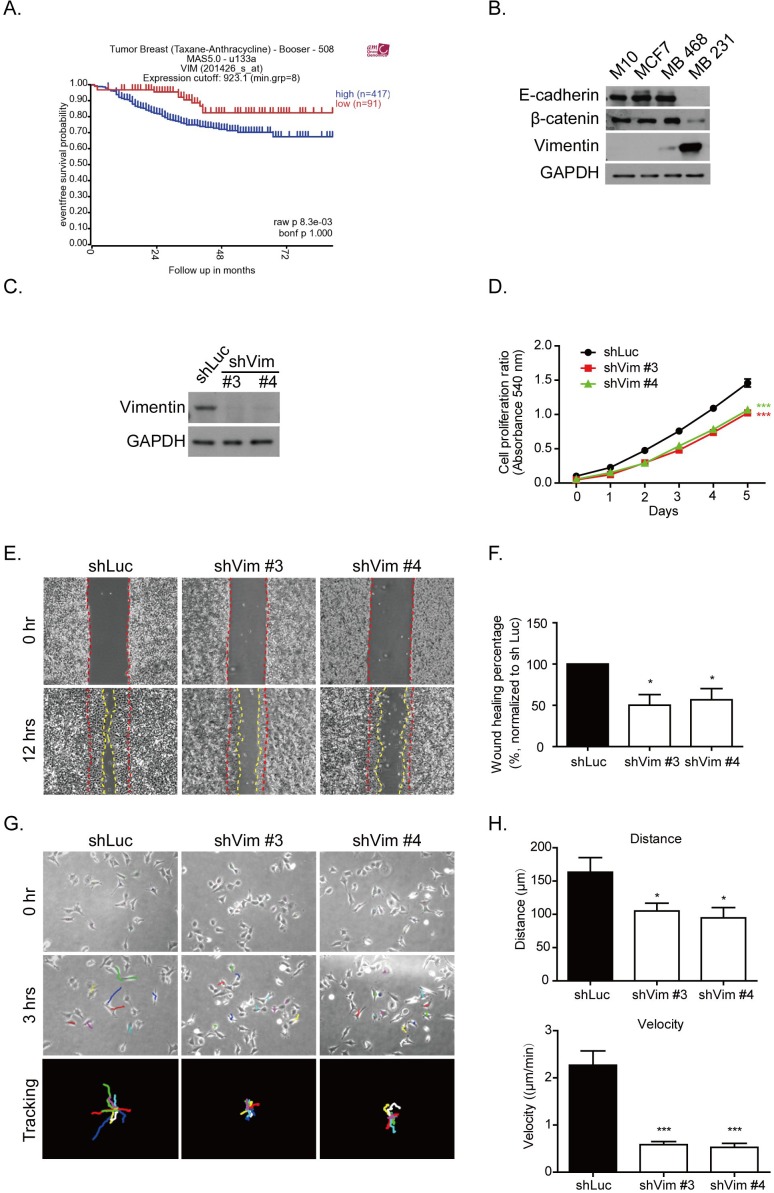
Vimentin expression contributes to breast cancer malignancy **A.** Kaplan-Meier analysis of event-free survival rate in breast cancer patients with vimentin mRNA expression. These patients were treated with chemotherapy drugs containing sequential Taxane- and Anthracycline-based regimens. The blue line indicates a high vimentin mRNA expression (*n* = 417), and the red line indicates a low vimentin mRNA expression (*n* = 91). The *p* value was 0.0083. Data were obtained from the R2 microarray public database (http://r2.amc.nl), Tumor Breast (Taxane-Anthracycline)-Booser-508 MAS5.0-u133a. **B.** Levels of several proteins from the normal breast epithelial cell line, M10, and three breast cancer cell lines, MCF7, MDA-MB 468, and MDA-MB 231, were analyzed using Western blotting. Cell lysate was collected after 24 hours of seeding. **C.** Protein levels of vimentin were examined in shRNA (shVim), expressing stable MDA-MB 231 #3 and #4 clones for various shRNA sequences. shLuc was performed as a control. **D.** Cell proliferation was analyzed using an MTT assay. Each bar represents mean ± SEM of three independent experiments. *** indicates *p* < 0.001 *vs.* shLuc control. E. Representative images of a wound healing assay. Control cells (shLuc) and vimentin knockdown stable clones (shVim #3, #4) were seeded for 18 hours until a monolayer was formed. The monolayer was then scraped using tips. Images were produced after scraping for 0 (space between red dashed lines) and 12 hours (space between yellow dashed lines). **F.** The quantitative data for wound healing is shown in the right panel. The wound healing percentage was normalized to shLuc. **G.** Representative images of shLuc and shVim #3, #4 time lapse tracking for 3 hours. Cells were seeded at low density for 18 hours, and cell migration was recorded for 3 hours. The upper panels showed the original cell position at 0 hours, and the middle panels showed the cell position after live cell tracking for 3 hours. Each colored line indicates the migration trace of each cell. All cell migration traces are placed in the same center in the tracking images. Cells measured for each condition: shLuc: *n* = 9; shVim #3: *n* = 11; and shVim #4: *n* = 12. **H.** Quantitative data of directional migration. Upper panels indicate the distances and lower panels show the velocity of cell migration. Each bar represents mean ± SEM. * indicates *p* < 0.05, and *** indicates *p* < 0.001 *vs.* shLuc control.

Alteration of gene expression levels is a common feature in tumorigenesis. Several types of cancer can become more invasive and malignant by undergoing the EMT process. Vimentin is one type of EMT protein marker, which is present in mesenchymal cells and involved in cancer progression [4, 7, 11, 15]. After we analyzed the tumor genomic microarray database R2 platform (http://r2.amc.nl), the results indicated that higher levels of vimentin mRNA contributed to the poor survival rate in patients after taxane and anthracycline chemotherapeutic treatment (raw *p* value = 0.0083) (Figure 1A). This result suggested the possible role of vimentin in cancer progression. To further prove this, we first investigated the protein levels of vimentin in the normal breast epithelial cell line, M10, as well as breast cancer cell lines with various levels of malignancy, such as MCF7, MDA-MB 468, and MDA-MB 231, which represented the cell lines at various stages: luminal (ER positive), basal-A (ER negative), and basal-B (ER negative and EMT phenotype) subtypes, respectively [32]. We analyzed the levels of EMT markers, such as E-cadherin, β-catenine, and vimentin. Figure 1B shows that M10, MCF7, and MDA-MB 468 exhibited high protein levels of E-cadherin and β-catenine, but lower levels of vimentin; MDA-MB 231 lost these epithelial markers but increased its levels of vimentin.

The vimentin shRNA was then expressed in MDA-MB 231 cells to generate stable knockdown clones. Two stable clones, denoted as #3 and #4, were selected by using various shRNA sequences. The knockdown efficiency is shown in Figure 1C. The cell proliferation was examined in these vimentin knockdown clones. After 5 days of MTT assay analysis, the proliferation of these vimentin knockdown cells was significantly reduced (Figure 1D). Cell migration ability was also examined using a wound healing assay. Figure 1E shows representative images of a scraping assay. At 0 hours, the wound spaces were generated between the red dashed lines as shown in the upper panels. After 12 hours, the migrated borders were generated, as indicated by the yellow dashed line in the lower panels (Figure 1E). The spaces between the red and yellow dashed lines were measured using ImageJ software. The quantitative data showed a significant decrease in cell migration in the vimentin knockdown clones (Figure 1F). We performed time-lapse tracking to observe single cell directional migration patterns. After 18 hours of seeding at low density, the cell migration routes were recorded for an additional 3 hours. As shown in Figure 1G, the upper panels show the cell position at 0 hours and the middle panels show the cell position after 3 hours. Each colored line in the middle and lower panels indicate the migration traces of each cell. We plotted all migration traces from various cells tail-to-tail, and the quantified results are shown in the lower panels. The time-lapse movies demonstrated that after vimentin knockdown, the cells generated large membrane extensions, membrane ruffling, and decreased their directional migration phenotype (Supplementary Movies 1-3). The quantitative results indicated that cell migration distances and velocity were significantly decreased in vimentin knockdown clones compared with the shLuc control (Figure 1H). These results indicated that the loss of vimentin decreased breast cancer cell proliferation and directional migration.

### Knockdown of vimentin increases membrane extension but loses focal adhesions

**Figure 2:**
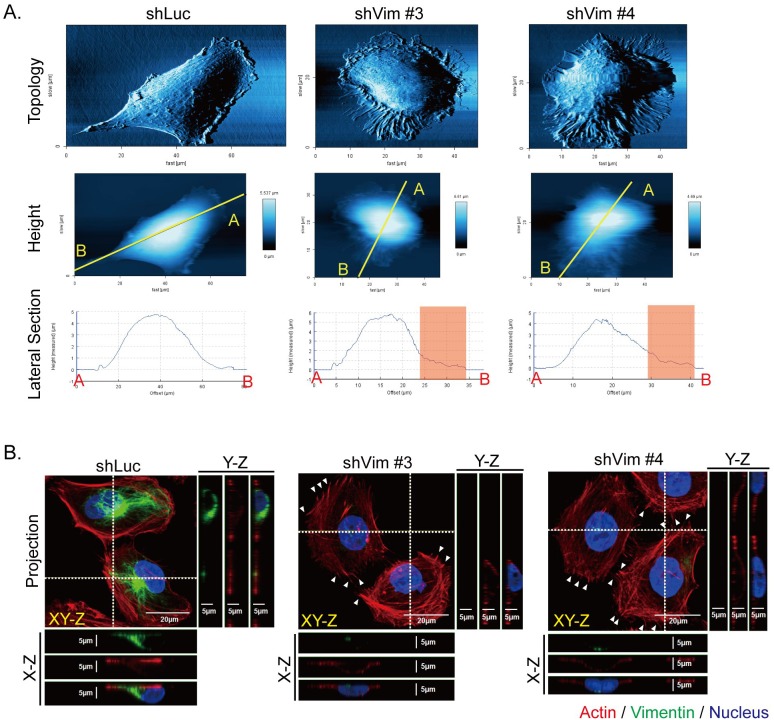
Knockdown of vimentin alters cell topology and cytoskeletal organization **A.** The cell surface topology of control (shLuc) and vimentin knockdown stable clones (shVim #3, #4) was scanned using AFM, as shown in the upper panels. The cell heights in the middle panels were evaluated after cell surface topology was scanned. The yellow lines from A to B on each image indicate the region of interest for the LV of the cell surface topology. The lateral sections of cell height were evaluated from A to B, as shown in the lower panels. The red boxes in the lateral sections indicate the membrane extension region in the cell periphery. **B.** Immunofluorescence images of control (shLuc) and sh vimentin stable clones (shVim #3, #4) were produced using confocal microscopy with series sections. The XY-Z images were stacked from various cell layers and projected into one image. The horizontal white dashed line in each image represents the X-Z section of each cell, and this X-Z section image is shown underneath the image. The vertical white dashed lines in each image represents the Y-Z section of cells in the image, and the Y-Z image is shown to the right of each image. Red: actin; green: vimentin; blue: nucleus. Scale bar: 20 μm. White arrows indicate protruding actin spikes.

To investigate the detail of the membrane extension and ruffling structures of the vimentin knockdown cells, the general cell surface topology was scanned using atomic force microscopy (AFM). The representative images show that the vimentin knockdown clones generated more membrane extensions and spikes than the control (shLuc) cells did (Figure 2A, upper panels). The lateral section was observed to determine cell height, which was similar in shLuc and vimentin knockdown clones (lower panels); however, the membrane extension region shown in the red boxes became rougher in vimentin knockdown cells (lower panels). These topological results indicated that the cytoskeletons underneath cell membranes may be reorganized. We applied immunostaining to observe the organization of actin and vimentin in the vimentin knockdown clones. In Figure 2B, the representative projected immunostaining images produced by confocal microscopy show the cytoskeleton distribution in shLuc and vimentin knockdown cells. In the lateral view (LV) of the X-Z and Y-Z sections of the shLuc cells, vimentin is located both on one side of the nucleus and on the top of the nucleus. F-actin was organized with a limited amount of regular stress fibers in cytosol in the control cells, whereas the actin filament formed irregular stress fibers in cytosol and spikes at the cell periphery in vimentin knockdown clones, indicated by the arrow in Figure 2B. These images reveal that a loss of vimentin resulted in reorganization of actin filament, which may help to rebalance disrupted cytoskeleton architecture.

**Figure 3:**
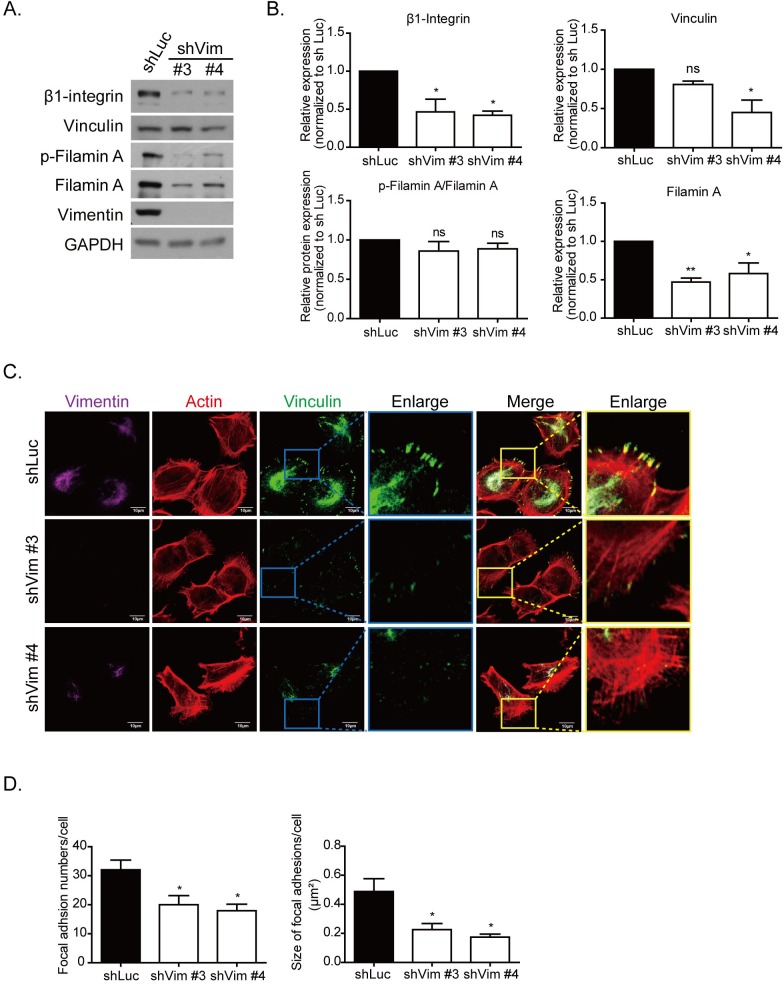
Knockdown vimentin decreases formation of focal adhesions **A.** Representative protein levels of focal adhesion complex proteins in control (shLuc) and vimentin knockdown stable clones were examined using Western blotting. GAPDH was performed as an internal control. **B.** Quantitative data of focal adhesion proteins. Levels of protein expression were normalized to GAPDH. Each condition was repeated at least three times. ns, *, and ** indicate *p* > 0.05, *p* < 0.05, and *p* < 0.01, respectively, as compared with shLuc. **C.** Representative fluorescence images of vimentin knockdown cells. Immunostaining of vimentin (purple), actin (red), and vinculin (green) are shown in separate channels. The blue box in vinculin and yellow box in merge images indicate regions of interest, and are further enlarged in the right panels. Scale bar: 10 μm. **D.** The quantitative results of focal adhesion number (left) and size (right) in each clone of 10 cells in **C.** were calculated. Each bar represents mean ± SEM. * indicates *p* < 0.05.

We then examined the protein levels of the focal adhesion complex protein by using Western blotting. As shown in Figure 3A, β1-integrin and vinculin were downregulated in the vimentin knockdown clones. The adaptor proteins in focal adhesion, such as filamin A and its phosphorylated form, were also downregulated in vimentin knockdown cells (Figure 3A, 3B). To investigate whether the localization of focal adhesion was affected in vimentin knockdown cells, immunofluorescence was used to stain vinculin, actin, and vimentin cytoskeletons, and the images were produced using confocal microscopy. The results indicated that the number and size of focal adhesion were smaller and the fluorescence intensity of vinculin staining was weaker in vimentin knockdown cells than in shLuc cells. The enlarged images further illustrate the reduced size and number of the focal adhesion in the cell periphery (Figure 3C). The quantitative results of focal adhesion size (right panel) and number (left panel) are also shown in Figure 3D. Vimentin knockdown caused the extension of cell membranes, reorganization of actin cytoskeletons, and the formation of spike structures; however, it reduced the maturation of focal adhesions.

### Microtubule is reoriented toward the top and bottom of the cell in vimentin knockdown cells

**Figure 4:**
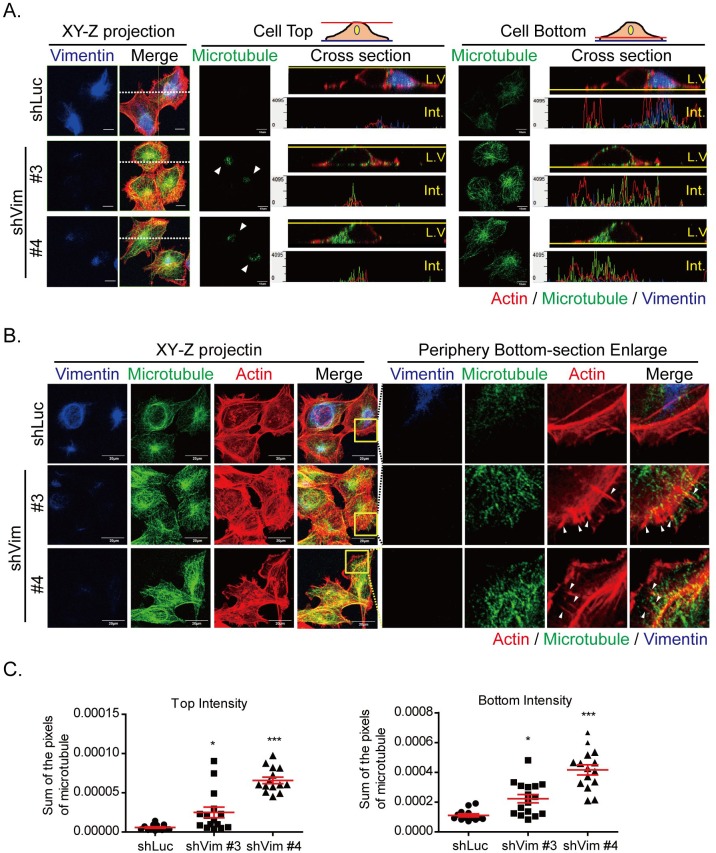
Microtubule is reoriented toward cell top and bottom in vimentin knockdown cells **A.** Photosectioning images of control (shLuc) and vimentin knockdown stable clones (shVim #3, #4) were produced using confocal microscopy. The images were separated into cell top and bottom for each clone. The left panels showed the XY-Z projection images of vimentin and merged images of three types of cytoskeleton. The horizontal white dash on the XY-Z projection images (left panels) indicates the LV section. Red: actin; green: microtubule; blue: vimentin. Scale bar: 10 μm. The fluorescence images were evaluated in each clone at cell top (middle panel) and cell bottom (right panel). In the middle panel, arrows indicate the microtubule staining at the cell top. The LV and section intensity at cell top or bottom are shown for each clone. **B.** Fluorescence images at the cell periphery of vimentin knockdown cells. Left panels indicate the XY-Z projected images of three types of cytoskeleton. Yellow boxes indicate the region of interest at the cell periphery. Scale bar: 20 μm. The regions of interest are enlarged, as indicated in the separate channels in the cell bottom sections. Red: actin; green: microtubule; blue: vimentin. Arrows indicate the actin spikes at the cell periphery. **C.** Quantitative results of the microtubule intensity of each clone at cell top (left panel) or bottom (right panel). * and *** indicate *p* < 0.05 and *p* < 0.001, respectively, compared with shLuc.

Because vimentin depletion resulted in the redistribution of focal adhesions and actin cytoskeletons, we used confocal microscopy (Figure 4A) to perform immunofluorescence staining at the top, bottom, cross section, and the Z-stack projection of cells to determine whether microtubule organization was also affected. In shLuc cells, microtubules were primarily distributed at the bottom; however, in vimentin knockdown clones, microtubules were reoriented at the top and bottom of the cells. The cross sections of cell tops and bottoms also demonstrated the redistribution of microtubules in the vimentin knockdown clones. The horizontal white dashed lines on the XY-Z projection images (left panels) indicates the LV section. The fluorescence intensity of the LV is shown in the panels illustrating the cell tops and bottoms. The microtubule was located at one side of the nucleus, as indicated by the LV and the fluorescence intensity of the cell top and bottom in the control shLuc cells. However, in vimentin knockdown clones, the microtubules were redistributed, covered the tops of nuclei and extended to the bottom of cells. The cell periphery region was also investigated (Figure 4B) to examine whether microtubules participated in membrane extension. The left panel individually shows the XY-Z projection images of cells with actin, microtubules, and vimentin staining. The projected images of microtubules in vimentin knockdown cells showed that microtubule filaments were disorganized, spread out, and extended toward the cell edges; this did not occur with the shLuc cells. Actin filaments with vimentin deficiency were also disorganized and formed around cells, particularly at the cell periphery. To observe the detailed cytoskeleton structures at the cell periphery, the yellow boxes in the merge panel were enlarged. The membrane extension areas revealed cytoskeleton distribution at the cell bottom (right panels). At the cell periphery, actin was the major type of cytoskeleton, and only several microtubules and vimentin were found in the shLuc cells (upper panels). Neither vimentin nor microtubules penetrated the membrane extension regions. However, microtubules in the vimentin knockdown cells extended to the cell membrane extension region, accompanied by actin spike structures (lower panels, white arrows). The quantitative results also demonstrated that the intensity of microtubules at cell tops and bottoms were significantly higher than that of shLuc cells (Figure 4C). In vimentin knockdown cells, actin formed strong filaments around cells and spike structures at the cell periphery. Microtubules were reoriented and extended to cell edges. The cytoskeleton redistribution tended to stabilize the cytoskeleton architecture within cells, which may result of the loss of mechanical strength of vimentin intermediate filaments.

### Knockdown of vimentin impairs cellular force generation

**Figure 5:**
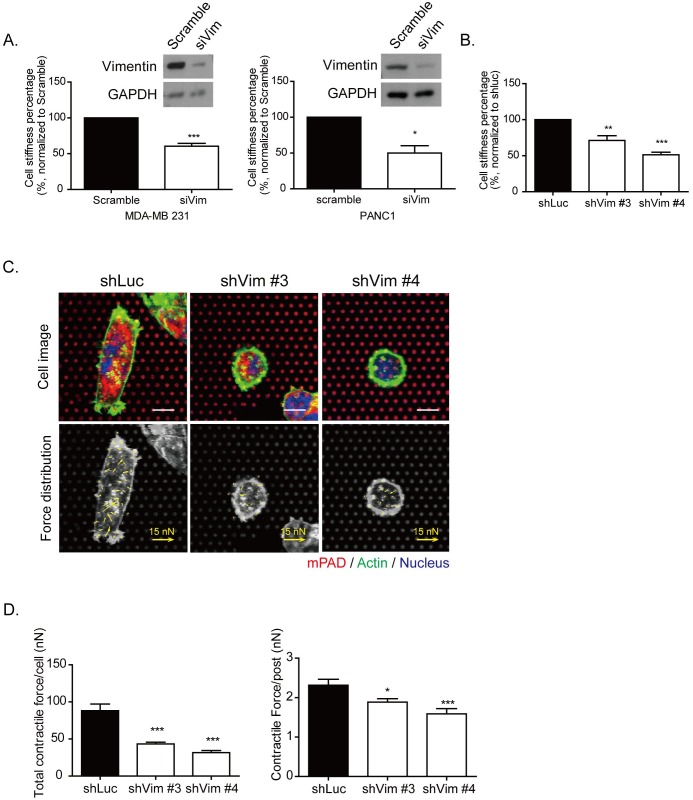
Knockdown vimentin impairs mechanical strength of cells **A.** The knockdown efficiency by siRNA specific to vimentin was examined by Western blotting, and cell stiffness was analyzed in MDA-MB 231 (left panels) and PANC1 (right panels) cells. The cell stiffness was examined using AFM in MDA-MB 231 and PANC1 cells expressing vimentin siRNA. The cell stiffness of si vimentin was normalized to scramble the control. Each bar represents mean ± SEM of three independent experiments. * and *** indicate *p* < 0.05 and *p* < 0.001, respectively, *vs.* the scrambled control. **B.** Cell stiffness was detected using AFM. The quantitative data of sh vimentin was normalized to shLuc. Each bar represents mean ± SEM of three independent experiments. ** and *** indicate *p* < 0.01 and *p* < 0.001, respectively, compared with the shLuc control. **C.** Representative images of vimentin knockdown clones seeded on mPAD. The upper panel showed the images of cells attached on the mPAD design. Each red dot represents a post top; green indicates actin and blue is the nucleus. Scale bar: 10 μm. The contractile force applied on each post and force distribution is indicated by the yellow arrows in the lower panel. The length of the arrows indicates the value of the contractile force. Scale bar of force arrows: 15 nN. **D.** Quantitative results of total contractile force in each cell (left panel) and the contractile force on each post (right panel). Each bar represents mean ± SEM. The calculated cell numbers of each clone were shLuc: *n* = 20; shVim #3: *n* = 18; and shVim #4: *n* = 16, collected from two independent experiments. * and *** indicate *p* < 0.05 and *p* < 0.001, respectively, as compared with shLuc.

In Figure 5A, siRNA of vimentin was applied separately in the MDA-MB 231 cells (left panel) and in a pancreatic cancer cell line of PANC1 cells (right panel). To measure cell stiffness, AFM indentation was performed to indent the stiffness on top of the nucleus of each cell. The quantitative results demonstrated that cell stiffness was significantly decreased in vimentin knockdown MDA-MB 231 and PANC1 cells. Similar results were also found in vimentin knockdown stable clones in MDA-MB 231 cells, as shown in Figure 5B. To detect cell contractile force, we performed microfabricated post-array detector (mPAD). The upper panels of Figure 5C show cells seeded on mPADs. The red dots indicate the top of the microposts, and the green fluorescence indicates the cell border of actin (upper panel). Cells attached onto the posts and generated the contractile force necessary for the posts to bend, causing the top of the posts to move, as indicated by the yellow arrows in the lower panels. Each yellow arrow indicates the contractile force generated on each post and demonstrates the force distribution within each cells. The quantitative results showed that total cell contractile force and the contractile force generated on each post were significantly reduced in vimentin knockdown cells (Figure 5D). The function of vimentin may contribute to organizing cytoskeleton distribution, as well as to mediating the mechanical properties of cells.

### Expressing vimentin in MCF7 cells increases cell stiffness and directional migration

**Figure 6:**
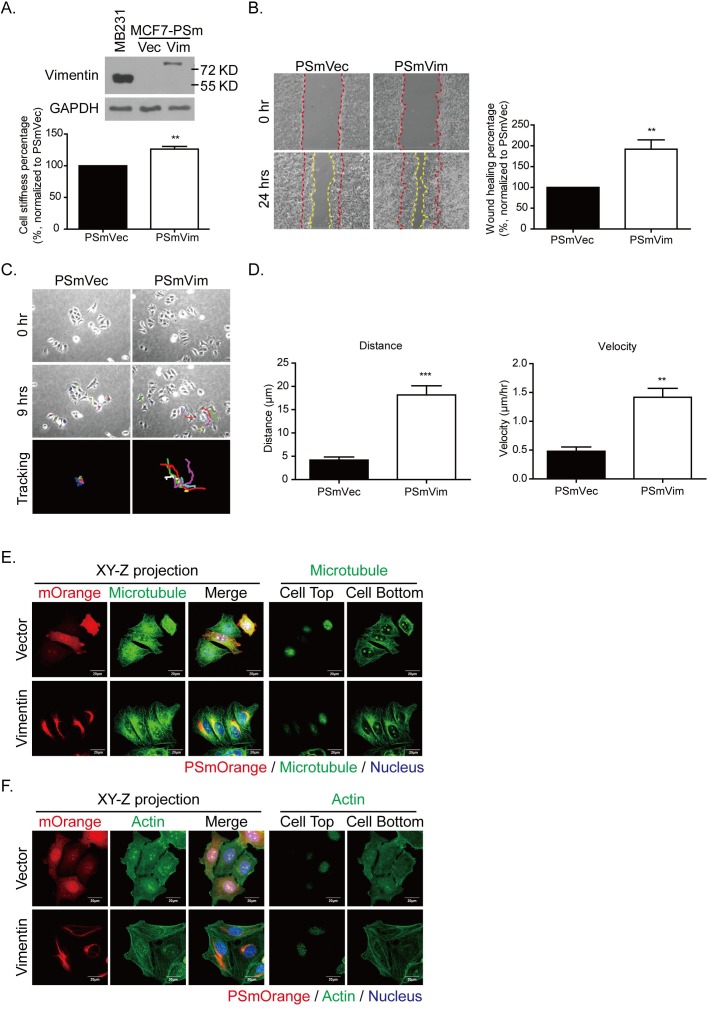
Expressing vimentin in MCF7 cells results in increased cell stiffness, cell motility, and directional cell migration **A.** Levels of vimentin overexpression in MCF7 cells were detected using Western blotting. MDA-MB 231 cells served as a positive control for vimentin protein expression. PSmOrange plasmid conjugated with vimentin was transfected into MCF7 cells. The molecular weight of vimentin was 57 KD, and the molecular weight of PSmOrange-vimentin shifted to 81 KD. Cell stiffness was analyzed using AFM indentation. Each bar represents mean ± SEM of 100 cells in each condition collected from three independent experiments. **B.** Representative images of wound healing assay. MCF7 cells were transfected with PSmVec as vector control and PSmVimentin. Cells were seeded for 18 hours until a monolayer was formed. The monolayer was then scraped using tips. Images were produced after scraping for 0 (space between red dashed lines) and 24 hours (space between yellow dashed lines). The quantitative data of wound healing is shown in the right panel. The wound healing percentage was normalized to PSmVec. **C.** Representative images of PSmVec and PSmVim time-lapse tracking for 9 hours. Cells were seeded at low density for 18 hours, and cell migration was recorded for an additional 9 hours. The upper panels showed the original cell position at 0 hours, and the middle panels showed the cell position after live cell tracking for 9 hours. Each line with a different color indicates the migration trace of each cell. All cell migration traces were placed in the same center in the tracking images. Cells measured for each condition: PSmVec: *n* = 24; PSmVim: *n* = 25. **D.** Quantitative data of directional migration. Left panels indicate distances, and right panels show the velocity of cell migration. Each bar represents mean ± SEM. ** indicates *p* < 0.01, and *** indicates *p* < 0.001 compared with the PSmVec control. **E.** and **F.** are the representative immunofluorescence images of vimentin overexpressing MCF7 cells. **E.** The left panels show the XY-Z projected images of overexpressing vimentin or vector control (red), microtubule (green), and merge images. The nucleus is blue. The right panel shows the microtubule distribution at the cell top and bottom for each condition. Scale bar: 20 μm. **F.** The left panels show the XY-Z projected images of overexpressing vimentin or vector control (red), actin (green), and merge images. The nucleus is blue. The right panel shows the actin distribution at the cell top and bottom for each condition. Scale bar: 20 μm.

To identify the role of vimentin in cytoskeleton architecture regulation, the low malignant breast cancer cell MCF7 was transfected with a PSmOrange vector that was carrying vimentin. Figure 6A shows the protein levels of vimentin overexpressed in MCF7 cells. When the PSmOrange was conjugated with vimentin, the molecular weight of the overexpressed vimentin (PSmVim) was higher (81 KD) than the initial vimentin (57 KD) (Figure 6A, upper panel). Cell stiffness was also increased significantly in overexpressed vimentin MCF7 cells (Figure 6A, lower panel). To evaluate whether the gain of vimentin facilitated cell motility, cell migration was evaluated using a wound healing assay. The cell migration rate was recorded for 24 hours (Figure 6B, left panel). MCF7 cells overexpressing vimentin migrated significantly faster than the control PSmVec cells did in terms of wound closing (Figure 6B, right panel). We then applied the time-lapse recording to identify whether vimentin overexpression contributed to cell directional migration in MCF7 harboring vimentin (Figure 6C, Supplementary Movie 4 and 5). After recording for 9 hours, the tracing routes were organized tail-to-tail (Figure 6C, lower panel). The quantitative results in Figure 6D showed that migration distance and velocity were significantly higher in PSmVim than in the control group.

The XY-Z projection (Figure 6E, left panel) shows that microtubules were distributed evenly in the control PSmVec cells, but were localized to one side of the nucleus, forming microtubule polarity and largely colocalized with overexpressed vimentin. To further evaluate the locations of microtubules, the cell top and bottom sections were analyzed separately (Figure 6E, right panel). From the bottom section, the PSmVim cells exhibited more polarized microtubules than the PSmVec cells did. In addition, the vimentin overexpressing cells formed more actin filamentous structures than the control PSmVec cells did (Figure 6F, left panel). Figure 6F (right panel) shows that the actin filaments were localized at the cell bottom. These results showed that the overexpression of vimentin in vimentin-negative cells can reorganize cytoskeleton architecture, which enhances the mechanical strength of cells and promotes microtubule polarity to elevate the ability for directional migration.

### Vimentin mediates slug expression to promote epithelial-mesenchymal transition phenotypes in cancer cells

**Figure 7:**
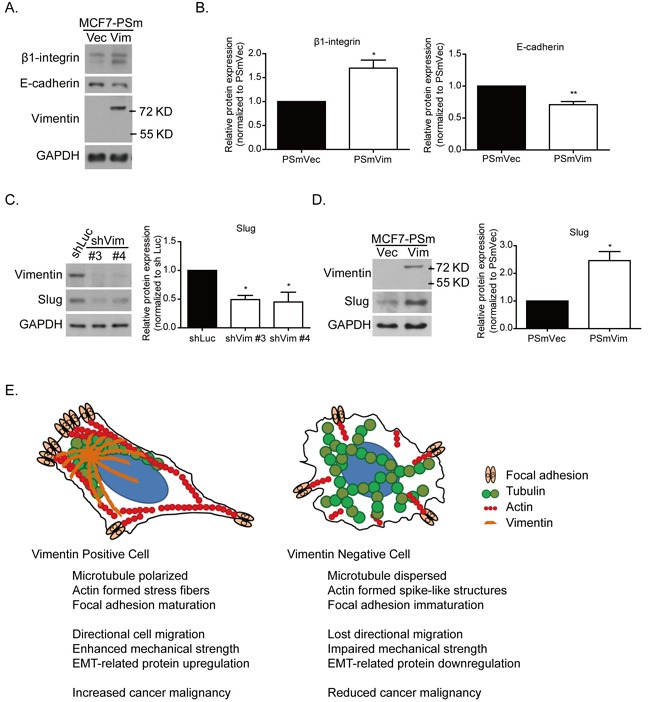
Vimentin mediates slug and EMT-related protein expression **A.** Protein expression of focal adhesion protein, such as β1-integrin and cell junction protein E-cadherin. **B.** Quantitative data of β1-integrin and E-cadherin. Levels of protein expression were normalized to GAPDH. Each condition was repeated at least three times. * and ** indicate *p* < 0.05 and *p* < 0.01, respectively, compared with PSmVec. The slug expression was examined in **C.** vimentin knockdown cells and **D.** vimentin overexpressing cells. The quantitative data of slug was normalized to GAPDH. Each condition was repeated at least three times. * indicates *p* < 0.05 compared with the control group. **E.** Systemic figure of tubulin (green), actin (red), and focal adhesion (light orange) orientation in vimentin (dark orange) positive and negative cells. In the vimentin positive cells, microtubules were polarized at the juxtanuclear region, and actin formed stress fiber structures at the cell bottom, accompanied by EMT-related protein expression. With the vimentin depletion, the microtubules were widely dispersed. Actin filaments formed spike-like structures at the cell periphery, accompanied by cell membrane extension. The focal adhesion was also reduced after vimentin knockdown. These changes caused a reduction in the mechanical strength of the cells and the ability for cell directional migration. This figure indicates the role of vimentin in EMT-related mechanoregulation and its contribution to cancer malignancy.

With higher motility, the PSmVim cells also exhibited significantly higher β1-integrin and lower E-cadherin protein expressions than PSmVec cells did (Figure 7A and 7B). These results implied that overexpressing vimentin causes the EMT in MCF7 cells [33]. Previous studies have shown that vimentin was upregulated in several types of cancer with poor prognoses, which is highly correlated with the upregulation of the EMT-related transcription factor, slug, and the downregulation of the adherent protein, E-cadherin [34, 35]. It was also showed that a vimentin-involved EMT phenotype was mediated using slug signaling [36]. Slug protein expression was analyzed in vimentin knockdown MDA-MB 231 cells (Figure 7C). The quantitative results showed that with vimentin depletion, slug protein expression significantly decreased. However, with overexpressed vimentin in MCF7 cells, the elevated vimentin expression was also accompanied by significantly increased slug protein expression (Figure 7D). These results showed that vimentin serves as a downstream effecter of the slug-mediated EMT process, as well as behaves as a regulator to control slug expression and promotes EMT-related cancer malignancy.

## DISCUSSION

Figure 7E shows the organization of cytoskeletons and the distribution of focal adhesions in cells expressing vimentin. In vimentin expressing cells (left panel), microtubules were polarized at one side of the cell nucleus, and primarily colocalized with vimentin distribution. This microtubule accumulation in the juxtanuclear region contributes to cell polarity and cell directional migration [37, 38]. However, in vimentin negative cells (right panel), the microtubules are reoriented and dispersed over the cell without creating any polarity. The knockdown of vimentin also results in the formation of immature focal adhesions, inducing additional cell protrusion and rapid membrane ruffling. It is also possible that F-actin is reorganized to form spike structures at the edge of a cell. These rearrangements of cytoskeletons and focal adhesions ultimately cause the reduction of directional migration and impaired cellular mechanical strength, reflected by the reduced cell stiffness and contractile force. Mechanically, vimentin mediates cytoskeleton architecture in the mechanical homeostatic state and maintains focal adhesion maturation. Through the colocalization of vimentin and microtubules, vimentin serves as a linker between actin and the microtubules, as well as a mediator to maintain cytoskeleton structure and mechanical force stability, which in turn contributes to cell polarity and motility. Vimentin also regulates the expression of the EMT-related transcription factor, slug, to further enhance EMT phenotypes and cancer malignancy. Therefore, the high level of vimentin observed in this study was positively correlated to the poor prognosis of breast cancer patients who had received cocktail chemotherapy (Figure 1A).

As an intermediate filament, vimentin acts as a type of mechanical cell sponge because of its flexible and elastic qualities, which help a cell to adapt and resist the compression and large deformation generated by microenvironments [31]. In addition to its assembling and disassembling properties that are shown by changing its soluble and insoluble ratio to sustain the mechanical properties of a cell [30], vimentin also contributes to the mechanical integrity of a cell by interacting with microtubules and actin cytoskeletons. This spatial organization of cytoskeletons contributes to cell tension and compression force to maintain cellular mechanical homeostasis, as indicated in the 6-strut tensegrity model [25]. These studies have focused on the mechanical role of vimentin in fibroblasts and endothelial cells. In the current study, our results revealed the role of vimentin in EMT-related cancer mechanotransduction. In vimentin depleted cancer cells, the cell malignancy was reduced and the mechanical strength was impaired. Without vimentin to coordinate the cytoskeleton webs into the appropriate types of architecture and stabilize focal adhesions, EMT-cancer cells were unable to generate appropriate cellular force and form cell polarity for further tumorigenesis. These findings suggest that vimentin contributed to EMT-related phenotypes by reorganizing cytoskeletons to form cell polarity, gain more motility, and enforce cellular tension generation. Therefore, in EMT cancer cells, vimentin maintained cell mechanical integrity by serving as a coordinator among cytoskeletons to further arrange the delicate cytoskeleton architecture and maintain the mechanical strength of cells. To maintain its cellular mechanical properties, vimentin relied on its elastic feature and fast dynamic polymerization. Vimentin also mediated cytoskeleton architecture by orienting microtubules and actin fibers, stabilizing focal adhesions, and then resulted in cancer cell force generation, migration, and malignancy.

### Vimentin mediates microtubule polarization to support cell directional migration

Previous studies have shown that microtubules are critical for vimentin polymerization and stabilization [39, 40]. It has been shown that vimentin directly interacts with microtubules; it forms the foci aggregation in the juxtanuclear region and colocalizes with γ-tubulin, which forms a microtubule organizing center [41]. Vimentin then polymerizes along the stabilized microtubules with the help of the microtubule-associated protein, kinesin [39, 40, 42]. However, vimentin also interacts indirectly with microtubules through intermediate filament-associated proteins (IFAPs), such as plectin [43]. The loss of plectin 1c in keratinocyte decreases microtubule dynamics, increases microtubule acetylation, forms smaller focal adhesions, and increases velocity, but loses cell directional migration [44]. These results indicate that the connections between vimentin and microtubules are critical to the maintenance of microtubule stability, which is similar to the results of this study regarding vimentin knockdown cells (Figure 4). In addition, overexpression of vimentin in MCF7 cells reorganized microtubule polarity and centralized microtubules to the juxtanucleus position (Figure 6E). These results indicated that although vimentin polymerized along microtubules and depended on the stability of microtubules, it also served as an organizer to mediate microtubule orientation. We propose that vimentin provides mechanical constraint strength at the juxtanucleus region to stabilize microtubule filamentous structures and directs the microtubule extension toward the cell periphery. In the case of vimentin depletion, microtubules thus spread and extend to cell edges, becoming more disorganized and leading to the loss of cell polarity and reduced cell directional migration.

### Vimentin sustains cellular force balancing

Actin cytoskeletons and focal adhesion maturation are an intracellular tension generation source, which is involved in cell shape maintenance [16, 45]. Vimentin connects mechanotransduction webs within cells, from focal adhesions to cytoskeletons, such as actin, microtubules, and nucleus cytoskeleton nesprins, and directly or indirectly through IFAPs, such as plectin [46-48], to modulate cell spreading and adhesion [49]. The tail domain of vimentin directly interacts with actin cytoskeletons to enhance the mechanical strength of cytoskeletal web [50]. In lung cancer cells, phosphorylated Rac1 GEF VAV2 colocalizes with FAK at focal adhesion sites and activates Rac1 signaling, allows FAK phosphorylation, and promotes cell attachment in vimentin-expressing cells [51]. Vimentin also interacts with the mechanosensor β1-intergrin cytoplasmic tail domain [52] to promote intergrin recycling with vimentin phosphorylation [53]. Our study demonstrated that vimentin-mediated focal adhesion maturation may act through β1-intergrin to stabilize peripheral membrane extension and generate cell mechanical strength. In addition, the adaptor protein filamin A was also downregulated in vimentin knockdown cells, suggesting that vimentin depletion impaired the coordination and mechanotransduction of the force generation network from focal adhesion signaling to the cytoskeletons inside the cell. The rough membrane surface scanned by AFM topology indicated that cytoskeletons underneath a cell membrane may undergo reorganization. Without vimentin to stabilize the intracellular mechanical networks, actin cytoskeletons were reorganized and formed spike-like structures at cell edges; they extended to the peripheral membrane but were unable to anchor on the unstable focal adhesions. Without mature focal adhesion and stable actin stress fibers, cells were not able to generate appropriate cellular tensions, resulting in reduced cell stiffness and impaired contractile force generation.

Previous studies have shown that fibroblast stiffness is reduced when vimentin is disrupted or deficient [25, 31], and similar findings were found in the current study. Because a part of the vimentin covered the top of the nucleus, this contributed to the strength of cell stiffness. We applied mPADs to measure the contractile force directly from the cell bottom [54, 55] of vimentin deficient cancer cells. The results indicated that cells without vimentin expression generated less contractile force on each single attached post, and further reduced the entire cell contractile force.

In general, when cells undergo the EMT process, their cytoskeletons are reorganized, accompanied by vimentin overexpression, and an increase in motility. However, how vimentin contributes to EMT-related cancer malignancy is unknown. In this study, we evaluated the role of vimentin for both the mechanic and tumorigenic approaches to breast cancer cells. Vimentin contributed to cytoskeleton organization and focal adhesion stability, which indicated that vimentin maintains cancer cell mechanical homeostasis. With the mechanical modulations generated by vimentin, the EMT-related cancer cells became increasingly organized to resist various stresses generated by the tumor microenvironment, and thus increased in malignancy. Moreover, vimentin was not only the downstream product of slug-mediated EMT signaling, but also a positive regulator to promote slug expression. With the increase in vimentin, slug expression was increased, and EMT-related gene expression was further elevated to further promote cancer malignancy. This study provided new insight into vimentin for EMT-related cancer mechanotransduction and tumor malignancy.

## MATERIALS AND METHODS

### Cell lines and cultures

The normal mammary human epithelial cell line M10 and three human breast cancer cell lines MCF7, MDA-MB 468, and MDA-MB 231 were kind gifts from Dr. Ming-Der Lai (National Chung Kung University, Taiwan) and were maintained in low glucose Dulbecco's modified Eagle's medium (DMEM, GIBICO). PANC1 was the pancreatic cancer cell line which was the kind gift from Dr. Shaw-Jenq Tsai (National Chung Kung University, Taiwan), and was maintained in Roswell Park Memorial Institute medium (RPMI 1640, GIBICO). Cells were cultured in the medium supplemented with 10 % FBS, 100 IU/ml penicillin and 100 μg/ml streptomysin, excepted PANC1 cells added with extra 1% L-glutamine (GlutaMAX^TM^-l, GIBCO). Cells were maintained at 37°C in a humidified 5 % CO_2_ incubator.

### Plasmids, shRNA, siRNA, and antibodies

Vimentin overexpressed plasmid was purchased from Addgene 31922: pVimentin-PSmOrange with full length of vimentin sequence insertion [56]. Plasmids of vimentin knockdown were purchased from National RNAi Core Facility Platform in Taiwan:

shLuc: TRCN0000072244, ATCACAGAATCGTCGTATGCA,
shVim #3: TRCN0000029119, GCTAACTACCAAGACACTATT,
shVim #4: TRCN0000029121, GCAGGATGAGATTCAGAATAT.

Cells were transfected by different shVim sequences in a cell population and then selected by Puromycin for two weeks to generate stable clones for further experiment. Vimentin overexpressing stable clones were transfected in a cell population and then selected by Neomycin, followed by sorting to purify the vimentin expressing cells.

siRNA were purchased from Thermo (L-003551-00-0005, ON-TARGETplus SMARTpool, Human Vim) combined four siRNA sequences together:

J-003551-06, UCACGAUGACCUUGAAUAA;
J-003551-07, GAGGGAAACUAAUCUGGAU;
J-003551-08, UUAAGACGGUUGAAACUAG;
J-003551-09, GGAAAUGGCUCGUCACCUU.

And the control scramble siRNA sequence was (D-001810-01-05, ON-TARGET plus Non-targeting siRNA #1): UGGUUUACAUGUCGACUAA

The primary antibodies used in the study, were as followed: β1-integrin (BD), GAPDH (Santa Cruz), vimentin (Millipore), vinculin (SIGMA), paxillin (BD), E-cadherin (BD), β-catenin (BD), slug (Cell Signaling), filamin A (Cell Signaling), and p-filamin A (Cell Signaling).

### MTT cell proliferation assay

Cells were seeded 2×10^3^ cells in 96 well plates with 100 μl medium. MTT solution was generated by Thiazolyl Blue Tetrazolium Bromide (Sigma) in MQ at 5 mg/ml. For cell proliferation evaluation, 10 μl MTT was added into 96 wells at indicated time. After incubating for four hours, medium was withdrawn and added 100 μl DMSO in each well for five minutes and then measured absorbance at 540 nm. Each cell condition was triplicated, and was repeated for three independent experiments.

### Western blots

Cells were rinsed with cold phosphate-buffered saline (PBS) and lysed in cold RIPA buffer (50 mM Tris-HCl, pH 7.4, 1% Triton X-100, 0.25% deoxycholate, 0.5% NP40, 0.1% SDS, 150 mM NaCl, 1 mM EDTA, 1 mM PMSF, 1 mM orthovanadate, 1 mM NaF, and Complete^TM^ (Roche)). Twenty μg proteins were resolved by 10% or 12.5% SDS-PAGE, followed by electroblotting onto nitrocellulose paper. For the slug protein examination, 50 μg proteins were applied. The blots were then blocked with 5% non fat dry milk in TBS-T, followed by incubating with specific primary antibodies. The immunocomplex was then detected using horseradish peroxidase-conjugated secondary antibodies. For visualization of the proteins, the fluorography was enhanced by chemi-luminescence (ECL) detection kit (GE Healthcare).

### Wound healing assay

Cells were seeded with 1×10^5^ cells in 6-well plate for 18 hours in normal culture medium. The p200 tips were applied for cell scraping and wound creating. After scraping, cells were incubated in low serum medium (1% FBS contained) for indicated time. In order to compare the wound healing percentage, the 0 hour images were taken right after wound creation by the inverted epifluorescence microscope (Nikon Ti-E H600L) with a 20X objective. Wound healing percentage was calculated at indicated time from taken images. The cell migration area was measured between dashed regions by Image J and normalized to control cells.

### Timelapse tracking

Cells were seeded with 3×10^4^ cells in 3 cm dishes in culture medium for 18 hours. Medium was then changed into low serum medium (1% FBS contained) before taking images. Each image was taken by Leica DM IRE2 inverted epifluorescence microscopy with a 20X objective. The vimentin knockdown cells were taken for 5 minutes interval and recorded for three hours duration. The vimentin overexpressed cells were taken for 15 minutes interval and recorded for 9 hours duration. Cell migration routes, distance, and velocity were analyzed by Image J software. The quantitative results were compared to shLuc control in MDA-MB 231 vimentin knockdown cells and PSmVec control in MCF7 vimentin overexpressed cells. Cells analyzed of each condition: shLuc: *n* = 9, shVim #3: *n* = 11, shVim #4: *n* = 12, PSmVec: *n* = 24, PSmVim: *n* = 25.

### Immunofluorescence imaging

Cells were rinsed three times with ice-cold PBS and fixed with 4% paraformaldehyde in PBS, permeablized with 0.5% Triton X-100 in PBS for 10 min, and followed by incubating in SuperBlock^®^ Blocking Buffer (Thermo). Samples were then incubated with specific first antibody at 4°C overnight. After extensive wash, samples were stained with secondary antibody by anti-mouse or anti-rabbit IgG conjugated with Alexa 488 nm (Invitrogen) or Alexa 594 nm (Invitrogen), phalloidin-TRITC (Sigma) or phalloidin-FITC (Sigma) and Hoechst 33258 (10 μg/ml) for 1 hour at room temperature. After that, samples were further washed by PBS and fixed to take images using an inverted scanning confocal microscope (FluoView FV-1000, Olympus) with a 60X, oil immersion objective lens or the inverted epifluorescence microscope (Nikon Ti-E H600L) with a 20X objective lens. Images were projected and analyzed by FV10-ASW 4.0 Viewer software (Olympus). Cell fluorescence intensity was quantified by Image J and FV10-ASW 4.0 software.

### Cell stiffness measurement and cell surface topology scanning by atomic force microscopy (AFM)

Cells were trypsinized and seeded on culture dishes and kept in a 6 cm-dish at the density of ~1×10^5^ cells/dish for 18 hours. Before measurement, medium was switched to CO_2_-independent medium (Invitrogen). A BioCell (JPK Instruments, Berlin, Germany) device allowed the measurements to be conducted at 37 ÐC. A diameter of 5 μm polystyrene bead was attached to the cantilever (MikroMasch, Tallinn, Estonia). Cantilevers were calibrated prior to each measurement and possessed a mean spring constants ranging from 0.01 to 0.03 nN/nm. Previous study revealed that the nuclear stiffness is considered as the most rigid site of endothelial cells [57]. Therefore, we applied the nucleus stiffness to indicate the whole cell stiffness. The force-distance curves were collected by landing the bead on the cell surface directly above the center of nucleus. The approaching speed of the cantilever was set at 1 μm/s and the relative set point was 1 nN. For each cell line, a minimum of 50 cells were analyzed in two to three independent experiments. Force-distance curves were processed using JPK IP software. For AFM measurement, the indentation surface is locally and with a specific tip shape. Cell was assumed incompressible and a Poisson's ratio of 0.5 was used. To extract the mechanical properties of cells from force-distance curves, the Hertz model were applied for the analysis of the data, as described previously [58]. For cell surface topology, cells were seeded at same density as for stiffness indentation. The AFM topology images were scanned by pyramidal tip (T1L450B, NANOSENSORS, US) in contact mode with 30-50 μm/sec tip scanning velocity. The scanning force was generally 0.8-1 nN for cells [58].

### Microposts array detector (mPAD) fabrication

To detect the intracellular contractility, we established mPAD force detecting system according to the methods published previously [55]. The processed wafers with specific features were kindly provided by Dr. Jianping Fu (University of Michigan). Briefly, to fabricate silicon micro-post array, the negative molds were first cured for 16 hours and then silanized overnight with 1H, 1H, 2H, 2H,-Perfluorodecyltrichlorosilane, 96% CF_3_ (CF_2_)_7_ (CH_2_)_2_SiCl_3_ (Alfa Aesar, Catalog: L16584) to prevent from permanently bonding to the negative molds. Liquid PDMS prepolymer was mixed at a 10:1 ratio and pour onto the negative master mold. Cover slips were pretreated in a plasma etcher and then directly stamped by negative molds with PDMS. The molds with mixed PDMS were further cured at 65°C for 16-18 hours. The mPADs were then peel off from the negative molds and the excess PDMS at the edge was cutoff. In order to facilitate the post feature, the CO_2_ critical dyer was further applied. For cell attachment, cured PDMS was cut into indicated size as a stamp to transfer ECM to mPAD post top for cell adhesion. Stamps were coated with 50 μg/ml fibronectin (BD) for one hour and then washed excessive fibronectin by MQ. With ozone surface activation, fibronectin was stamped from PDMS stamps to post top of mPADs. Cells were seeded on mPAD for 16 hours and fixed for further analysis. DiI (Invitrogen, D38886) was then applied to label microposts for cellular force quantification [55].

### Statistics

All data were presented as mean ± SEM of at least 2 to 3 independent experiments. Comparison was performed with two-tailed student's t-test or One-Way ANOVA. P<0.05 was taken to be statistic significant.

## SUPPLEMENTARY MATERIAL MOVIES












