# Supplementary material for: Vimentin contributes to epithelial-mesenchymal transition cancer cell mechanics by mediating cytoskeletal organization and focal adhesion maturation
Source: Oncotarget. 2015 Apr 18;6(18):15966–83. doi: 10.18632/oncotarget.3862 (PMC4599250; doi:10.18632/oncotarget.3862)
Supplement: Supplementary file 1 [file oncotarget-06-15966-s001.pdf]

# Vimentin contributes to epithelial-mesenchymal transition cancer cell mechanics by mediating cytoskeletal organization and focal adhesion maturation

**Supplementary Material**

Supplemental movies

Cells were seeded with $3x10^4$ cells in 3 cm dishes in culture medium for 18 hours. Medium was then changed into low serum medium (1% FBS contained) before taking images. Each image was taken by Leica DM IRE2 inverted epifluorescence microscopy with a 20X objective. The MDA-MB 231 and vimentin knockdown cells were taken every 5-minute and recorded for 3 hours duration. The MCF7 and vimentin overexpressed cells were taken every 15-minute and recorded for 9 hours duration. Cell migration routes, distance, and velocity were analyzed and by Image J software. Cell migration routes were labeled with different colors of each traced cells. The quantitative results were compared to sh Luc control in MDA-MB 231 vimentin knockdown cells and PSmVec in MCF7 vimentin overexpressed cells Cells analyzed of each condition: shLuc: n=9, shVim #3: n=11, shVim #4: n=12, PSmVec: n=24, PSmVim: n=25.

Movie 1: Live recording of sh Luc MDA-MB 231 cells

Movie 2: Live recording of sh vim #3 MDA-MB 231 cells

Movie 3: Live recording of sh vim #4 MDA-MB 231 cells

Movie 4: Live recording of PSmVec MCF7 cells

Movie 5: Live recording of PSmVim MCF7 cells
